# Consensus Statements from the Diabetologists & Endocrinologists Alliance for the Management of People with Hypertension and Type 2 Diabetes Mellitus

**DOI:** 10.3390/jcm12103403

**Published:** 2023-05-11

**Authors:** Peter Chun-Yip Tong, Susanna Chi-Pun Chan, Wing-Bun Chan, Kelvin Kai-Leung Ho, Godwin Tat-Chi Leung, Stanley Hok-King Lo, Gary Yiu-Kwong Mak, Tak-Sun Tse

**Affiliations:** Diabetologists & Endocrinologists Alliance, Hong Kong SAR, China

**Keywords:** albuminuria, angiotensin receptor blockers, hypertension, type 2 diabetes mellitus

## Abstract

Hypertension and type 2 diabetes mellitus (T2DM) are important, intertwined public health issues. People with both conditions face significantly elevated risks of cardiovascular (CV) and renal complications. To optimize patient care, a multidisciplinary expert panel met to review recent evidence on optimal blood pressure (BP) targets, implications of albuminuria, and treatment regimens for hypertensive patients with T2DM, with the aim of providing recommendations for physicians in Hong Kong. The panel reviewed the relevant literature, obtained by searching PubMed for the publication period from January 2015 to June 2021, to address five discussion areas: (i) BP targets based on CV/renal benefits; (ii) management of isolated systolic or diastolic hypertension; (iii) roles of angiotensin II receptor blockers; (iv) implications of albuminuria for CV/renal events and treatment choices; and (v) roles and tools of screening for microalbuminuria. The panel held three virtual meetings using a modified Delphi method to address the discussion areas. After each meeting, consensus statements were derived and anonymously voted on by every panelist. A total of 17 consensus statements were formulated based on recent evidence and expert insights regarding cardioprotection and renoprotection for hypertensive patients with T2DM.

## 1. Introduction

Hypertension is an important public health issue, affecting an estimated 26% of the global population (972 million people). This figure is expected to increase to 29% by the year 2025 [1]. According to the Hong Kong Population Health Survey 2014/2015, 27% of the population aged ≥15 years had hypertension, with 47.5% being undiagnosed before the survey [2]. Untreated hypertension is associated with a risk of atherosclerotic disease in 30% of patients and organ damage in 50% of patients within 8–10 years after onset; the risks may increase further in patients with comorbidities (e.g., chronic kidney disease [CKD]) [3]. It was estimated that the mortality rates for both ischemic heart disease and stroke are doubled for every 20-mmHg systolic or 10-mmHg diastolic increase in blood pressure (BP) above 115/75 mmHg [4].

Type 2 diabetes mellitus (T2DM), another major health problem worldwide, is often intertwined with hypertension, which is 1.5–3 times more common in people with T2DM than in people without T2DM [5]. In Hong Kong, a territory-wide study found that hypertension was the most common coexisting chronic illness among people with T2DM, with a prevalence of 57.6% [6]. Hypertension and T2DM are both risk factors for cardiovascular (CV) disease, stroke, progression of renal disease, and diabetic retinopathy [4]. In the Framingham Heart Study, people diagnosed with hypertension and T2DM were found to have a 72% increase in the risk of all-cause death and a 57% increase in the risk of any CV event [7]. A study of 6800 adults with hypertension and T2DM from 10 Asian countries found that 18.8% and 39.8% had macroalbuminuria and microalbuminuria, respectively [8]. Patients with diabetic nephropathy and elevated BP are associated with a high risk of developing end-stage renal disease (ESRD) [9].

For the treatment of hypertension in people with T2DM, angiotensin-converting enzyme inhibitors (ACEIs) and angiotensin receptor blockers (ARBs) have been recommended as first-line agents across international guidelines [10,11,12,13], on the basis of their BP-lowering efficacy as well as benefits of reducing diabetic nephropathy progression and increasing insulin sensitivity [14,15]. Although the efficacy and safety of these two drug classes are often considered similar, a recent large head-to-head analysis suggests that, for first-line antihypertensive treatment, ARBs may offer similar CV protection with an improved safety profile compared with ACEIs [16]. With respect to BP targets, the CV and renal benefits of achieving <140/90 mmHg have been well-established [17]. However, whether further BP lowering provides extra benefits, particularly in high-risk populations (e.g., with T2DM or of an older age), remains dubious [18,19,20]. Another area of interest is how managing albuminuria, in addition to BP, reduces the risks of CV and renal complications in people with hypertension and T2DM. 

To optimize the management of hypertension in people with T2DM, an expert panel was convened to review the roles of ARBs, effects of intensive BP lowering, and implications of albuminuria control, from the perspective of reducing the risks of acute coronary syndromes, stroke, heart failure, CV death, progression of CKD, and diabetic retinopathy. 

## 2. Materials and Methods

Members of the multidisciplinary panel were invited to participate by the Diabetologists and Endocrinologists Alliance on the basis of their interest and experience in managing patients with hypertension and T2DM in Hong Kong. The panel included three cardiologists, three endocrinologists and two nephrologists. 

They discussed the literature, including studies conducted in Hong Kong and the Asia-Pacific region, and their clinical experience in the following areas related to the management of hypertension in patients with T2DM: (i) BP targets based on CV and renal benefits; (ii) management of isolated systolic (ISH) or diastolic (IDH) hypertension; (iii) roles of ARBs; (iv) implications of albuminuria for CV/renal events and treatment choices; and (v) roles and tools of screening for microalbuminuria. 

The expert panel commissioned a medical writing agency to conduct a literature search. The PubMed database was searched for clinical trials (including sub-analyses), meta-analyses, and observational studies, as well as clinical guidelines related to the above areas, for the publication period between January 2015 and June 2021, using the keyword ‘hypertension’ combined with the following additional keywords: ‘albuminuria’; ‘angiotensin receptor blocker’; ‘Asian’; ‘blood pressure’; ‘cardiovascular disease’; ‘Chinese’; ‘chronic kidney disease’; ‘diabetes’; ‘diabetic nephropathy’; ‘diabetic retinopathy’; ‘Hong Kong’; ‘proteinuria’; and ‘screening’. The agency screened the titles and abstracts to identify the relevant papers that addressed the discussion areas. The full text of each screened paper was then scrutinized for relevancy by the expert panel. 

The panelists held a total of three virtual meetings using a modified Delphi method [21] to discuss the above areas. After each meeting, consensus statements were derived and anonymously voted on by every panelist. A consensus statement was accepted only if ≥80% of the panelists selected ‘accept completely’ or ‘accept with some reservation’ among the other options, which included ‘accept with major reservation’, ‘reject with reservation’, and ‘reject completely’.

## 3. Results

A total of 17 consensus statements (Table 1) were formulated and unanimously accepted, with the rationales detailed in the following sections.

## 4. Discussion

Part I: BP targets based on CV and renal benefits.

Statement 1: Home BP, in addition to office BP, should be considered for guiding treatment; home BP is generally considered to be 5 mmHg lower than office BP.

The panelists noted that, although office BP, which has been used widely in studies, remains the standard reference for prescribing antihypertensive treatment, the white-coat effect is common among real-life patients, whose office BP is often higher than their home BP. Another consideration of the panel was that multiple studies have shown the prognostic value of home BP in predicting preclinical organ damage, CV mortality and CV events [22,23]. Therefore, the panel agreed that, along with office BP, home BP is worth considering in treatment decision-making. To obtain a more reliable average home BP, patients should be asked to use an arm-type BP monitor (calibrated against a clinic BP monitor) in the morning and evening on a regular basis [12]. To address the potential bias of self-recorded home BP, researchers are exploring the use of telemedicine technology to assess the quality of home BP data and thus improve the prevention of hypertension [24,25]. The panel discussed that, as per the European Society of Cardiology (ESC) and the European Society of Hypertension (ESH), home BP is generally considered to be 5 mmHg lower than office BP (e.g., office BP of 140/90 mmHg corresponds to home BP of 135/85 mmHg) [12].

Statement 2: In patients with T2DM, hypertension is diagnosed when office BP is ≥140/90 mmHg and home BP is ≥135/85. 

After reviewing the available evidence, the panel recommended that people with T2DM who have a systolic BP (SBP) of ≥140 mmHg should be diagnosed as hypertensive, and thus requiring antihypertensive drug treatment. A territory-wide cohort study of people with T2DM and hypertension in Hong Kong showed that an SBP of ≥140 mmHg (or <125 mmHg) was associated with elevated risks of both CV diseases and all-cause mortality [26]. In a meta-analysis of 49 studies that included more than 73,000 people with T2DM, antihypertensive drug treatment was shown to provide survival and CV benefits among those with a baseline SBP of ≥140 mmHg. For those with SBP > 150 mmHg, relative risks for all-cause mortality, CV death, myocardial infarction (MI), stroke, and ESRD were 0.89, 0.75, 0.74, 0.77, and 0.82, respectively, while those with SBP 140–150 mmHg had relative risks of all-cause mortality, MI, and heart failure of 0.87, 0.84, and 0.80, respectively. However, those with a baseline SBP of < 140 mmHg had an increased risk of CV death (relative risk, 1.15) with further antihypertensive treatment [27]. The panel noted that, although there was a lack of extensive studies that had used diastolic BP (DBP) as the primary measure to assess the effects of antihypertensive treatment on survival and CV outcomes, Western and Chinese guidelines have generally considered a BP of ≥140/90 mmHg as the threshold for hypertension in people with T2DM [12,13,28,29].

Statement 3: In patients with T2DM and hypertension on antihypertensive drug treatment, the targets should be <130/80 mmHg for office BP and <125/75 mmHg for home BP. 

The panel discussed the CV benefits of the BP treatment target of <130/80 mmHg in patients with T2DM and hypertension in the clinical setting. A meta-analysis of 123 BP-lowering trials showed that every 10-mmHg reduction in SBP significantly lowered the risk of major CV events, coronary heart disease, stroke, heart failure, and all-cause mortality by 20%, 17%, 27%, 28%, and 13%, respectively, with the size of the effects being comparable across high-risk populations, including those with T2DM, CV disease, coronary heart disease, stroke, heart failure and CKD, as well as different SBP groups, including those with an SBP of <130 mmHg [30]. In a more recent meta-analysis of studies on antihypertensive treatment in people with T2DM, intensive BP lowering, i.e., attaining an SBP of <130 mmHg from a baseline of <140 mmHg, was shown to further reduce the risk of all-cause mortality [31].

Statement 4: In patients with MI, other acute coronary syndromes, or stroke, the targets should be <130/80 mmHg for office BP and <125/75 mmHg for home BP.

Based on the literature, the panel recognized that a BP target of <130/80 mmHg remains suitable for patients with prior CV events. In the STEP randomized controlled trial of Chinese patients aged ≥60 with hypertension (19% with T2DM), the intensive treatment group (mean SBP, 127.5 mmHg) had reduced risks of stroke, acute coronary syndrome, acute decompensated heart failure, coronary revascularization, atrial fibrillation, and CV death (hazard ratios [HRs], 0.67, 0.67, 0.27, 0.69, 0.96, and 0.72, respectively) compared with the standard treatment group (mean SBP, 135.3 mmHg) [32]. The available evidence, as well as clinical guidelines [10,11,12,13,29,33], support treatments lowering BP to <130/80 mmHg among people with T2DM and hypertension, regardless of the presence or absence of prior CV events.

Statement 5: In patients with heart failure or CKD, the targets should be <130/80 mmHg for office BP and <125/75 mmHg for home BP.

Heart failure is an important comorbidity that may worsen prognosis in patients with hypertension, T2DM, or even prediabetes [34,35]. The panel noted that studies on BP targets for people with hypertension and a history of heart failure or CKD remain limited. A post hoc analysis of SPRINT showed that targeting SBP < 120 mmHg, compared with <140 mmHg, significantly reduced the risk of acute decompensated heart failure across several key subgroups, including patients who had prior CV disease or CKD [36]. However, the panel discussion stated that aggressive BP lowering may cause adverse outcomes, considering a reverse J-curve relationship between BP and all-cause mortality among patients with heart failure [37]. In a propensity-score–matched observational study of hospitalized patients with heart failure with preserved ejection fraction, a discharge SBP < 120 mmHg was significantly associated with higher risks of all-cause mortality and readmission for heart failure compared with a discharge SBP ≥ 120 mmHg [38]. Consistent with current guidelines, the panel recommended a target BP of 130/80 mmHg for patients with heart failure, considering the lack of concrete evidence supporting SBP < 120 mmHg [37,39].

Likewise, the panel recommended SBP < 130 mmHg, rather than < 120 mmHg, for patients with CKD [10,11,12,13]. Although a post hoc analysis of SPRINT demonstrated that, among patients with CKD and hypertension, targeting SBP < 120 mmHg, compared with <140 mmHg, reduced rates of major CV events and all-cause death [40], targeting SBP < 120 mmHg for patients with CKD remains controversial because of the absence of robust evidence supporting the benefit-risk profile of the practice in the majority of CKD populations [41]. 

Statement 6: In patients aged ≥ 80 years, the targets may be <140/90 mmHg for office BP and <135/85 for home BP. 

The panel considered that the BP target for older people with hypertension remains far from certain. A recent large meta-analysis [42] and an age-stratified analysis of the SPRINT trial [43] showed that the efficacy of intensive SBP lowering (<120 mmHg) for reducing the risk of a composite of major CV events was consistent across different age categories spanning <65 years to ≥80 years. The analysis of SPRINT also showed that the safety of intensive SBP lowering was not modified by age, whether tested categorically or continuously [43]. In the STEP study of Chinese people with hypertension aged from 60 to 80 years, intensive SBP lowering (mean, 127.5 mmHg) was associated with a lower incidence of a composite of major CV events, but a higher incidence of hypotension, compared with standard SBP control (mean, 135.3 mmHg) [32]. The panel noted that, although these data appear to support an SBP target of <120–130 mmHg in older patients with hypertension, the inclusion of only a small proportion (6–12%) of participants aged ≥80 limits the generalizability of the data. Another concern of the panel is the tolerability of treatment—the risk of postural hypotension related to intensive BP control should be a source of caution with older patients. In line with the ESC/ESH guidelines [12] and the International Society of Hypertension [13], the panelists considered that a BP target of <140/90 mmHg is acceptable in people with hypertension (with or without T2DM) aged ≥80.

Part II: Management of ISH or IDH.

Statement 7: Both ISH (defined as SBP ≥ 140 mmHg and DBP < 90 mmHg) and IDH (defined as SBP < 140 mmHg and DBP ≥ 90 mmHg) should be treated to reduce the risks of MI, stroke, heart failure, and CV mortality.

Based on the current evidence, the panel agreed that both ISH and IDH, as defined above, are associated with an increased risk of cardiovascular events and thus require antihypertensive treatment [12]. In a cohort of 1.3 million American adults in a general outpatient population, both ISH and IDH (HRs/unit increase in z score, 1.18 and 1.06) influenced the risk of a composite of CV events (i.e., MI, ischemic stroke, or hemorrhagic stroke) [44]. 

In a Korean nationwide health screening database that included 6.4 million adults aged 20–39, stage 1 ISH (SBP 130–139/DBP < 80 mmHg), IDH (SBP < 130/DBP 80–90 mmHg), and systolic–diastolic hypertension (SBP 130–139/DBP 80–90 mmHg) were all associated with a higher risk of a composite of CV events (i.e., MI, stroke, heart failure, or CV death) compared with BP of <120/<80 mmHg (HRs, 1.36, 1.32, and 1.67, respectively) [45]. 

In a systematic review and meta-analysis that included ~490,000 individuals [46], IDH (SBP < 140/DBP ≥ 90 mmHg) was significantly associated with elevated risks of composite CV events, CV mortality, all strokes, and hemorrhagic stroke, compared with a BP of <140/<90 mmHg (HRs, 1.28, 1.45, 1.44, and 1.64, respectively). Subgroup analysis further showed that younger (mean age, 55 years) or Asian patients with IDH were significantly associated with a higher risk of composite CV events. 

In the PROGRESS randomized placebo-controlled trial, antihypertensive treatment with perindopril reduced the relative risk of major vascular events by 27%, 28%, and 32% among patients with ISH, IDH, and systolic–diastolic hypertension, respectively [47]. Regardless of the type of hypertension, BP-lowering treatment provides a consistent level of vascular protection. 

Part III: Roles of ARBs

Statement 8: In patients with T2DM and hypertension, ARBs are the preferred antihypertensive drug regimen, considering their proven efficacy in reducing BP, as well as urine protein levels, the risk of ESRD, and progression of nephropathy. 

The panel endorsed the well-established BP-lowering efficacy of ARBs. A meta-analysis has demonstrated that ARBs, as a class, were more effective than placebo in long-term BP lowering, and were associated with a 21% reduction in the risk of stroke, compared with other antihypertensives, in patients with essential hypertension [48]. A systematic review showed that most ARBs at the lowest recommended doses can effectively reduce BP [49]. In a real-world study of 170,000 adults who received antihypertensive medications for ≥9 months [50], those on ARB-based dual therapy had a significantly higher BP goal attainment rate than did those on non–ARB-based dual therapy (39% vs. 31%; *p* = 0.004). ARB-based therapy was also associated with significantly fewer CV events compared with ACEI- or calcium channel blocker (CCB)-based therapy (4.3% vs. 7.0% and 11.0%, respectively; *p* < 0.001). 

The panel noted that, for hypertensive patients with T2DM, ARBs provide extra renal benefits in addition to BP reduction. A meta-analysis showed that ARBs were similarly effective to ACEIs in reducing urinary protein excretion in hypertensive patients with and without T2DM [51]. Another meta-analysis indicated that ARBs significantly reduced the risks of ESRD and the doubling of the serum creatinine level by 23% and 40%, respectively, among T2DM patients with macroalbuminuria or microalbuminuria [52]. In a randomized trial of patients with T2DM, ARBs were associated with significantly reduced urinary excretion of angiotensinogen in participants overall, and lower albuminuria in participants with high–normal albuminuria, compared with treatment with aliskiren, a direct renin inhibitor [53]. 

Statement 9: For the treatment of hypertension, ARBs are preferred over ACEIs, considering the frequent side effect of dry cough associated with ACEIs, and the lack of evident differences in the BP-lowering efficacy between ARBs and ACEIs.

Because both act on the renin–angiotensin system, ACEIs and ARBs have been considered similar drug classes and recommended equally as first-line antihypertensive medications in international guidelines. However, the panel emphasized that, based on local and overseas experiences, ACEIs appear to be less well-tolerated, primarily due to the side effect of dry cough. In a survey of Hong Kong patients with hypertension or heart failure, persistent cough was reported in 44% of those on ACEIs compared with 11.1% of those on other antihypertensive agents [54]. A recent multinational cohort study of patients who initiated antihypertensive monotherapy with ACEIs or ARBs showed that, while there were no significant differences in the risks of acute MI, heart failure, stroke, or composite CV events between the two drug classes, ACEIs were associated with significantly higher risks of cough, angioedema, pancreatitis, and gastrointestinal bleeding [16]. Consistent with the literature, the panel noted that ARBs are preferred over ACEIs due to a better tolerability profile in clinical experience. Notably, use of ACEIs and ARBs has been associated with hyperkalemia and increased creatinine levels, especially in patients with renal impairment [55,56]. Before and shortly after initiation of these medications, monitoring of serum potassium level and estimated glomerular filtration rate (eGFR) should be considered. 

Statement 10: Combination therapy with an ARB and a CCB can be considered for patients with inadequate BP control. 

The panel considered ARBs to be the preferred antihypertensive medication for patients with hypertension and T2DM on the basis of their BP-lowering efficacy and renal benefits. However, they noted that real-life patients often require dual therapy to optimize BP control. International guidelines have recommended that, on top of an ARB, a CCB or a thiazide diuretic can be added in patients with inadequate BP control [11,12]. Notably, the panel preferred CCBs. In the ACCOMPLISH randomized trial of patients with hypertension and at high risk of CV events, the ACEI–CCB combination (benazepril + amlodipine) was associated with significantly lower risks of a composite of CV events (9.6% vs. 11.8%; HR, 0.80) and development of CKD (2% vs. 3.7%) compared with the ACEI–diuretic combination (benazepril + hydrochlorothiazide) [57]. The panel discussion stated that, extrapolated from these results, an ARB–CCB combination might be more beneficial than an ARB–diuretic combination. 

Statement 11: Regardless of the presence or absence of T2DM or hypertension, albuminuria should be treated as early as possible, considering the significant and continuous association between the degree of albuminuria and the subsequent risks of major CV events, ESRD, and proliferative retinopathy; these risks are further elevated in hypertensive patients with T2DM and albuminuria.

The panel reviewed a number of studies on the relationship between albuminuria and CV events. Data from Western populations showed that, for every 0.4-mg/mmol increase in the urine albumin-to-creatinine ratio (UACR), the risk of a composite of CV events (i.e., MI, stroke, or CV mortality) increased by 5.9%, regardless of the diagnosis of T2DM [58]. A meta-analysis of cohort studies showed that microalbuminuria (urinary albumin 30–300 mg/d) and macroalbuminuria (urinary albumin > 300 mg/d) increased the risk of developing coronary heart disease by 50% and 200%, respectively, compared with normoalbuminuria, irrespective of other risk factors including T2DM [59]. In studies of Chinese populations, elevated UACRs were associated with significantly increased risks of hospitalization for heart failure, CV death, and all-cause mortality [60,61,62]. A study of Hong Kong patients with T2DM in the primary care setting demonstrated that the UACR was positively and linearly associated with risks of CV events and all-cause mortality [63].

CKD progression is often defined by change in eGFR, while the panel noted that the risk implications of change in albuminuria have increasingly been observed. Indeed, a UACR of >30 mg/g present for >3 months indicates the development of CKD, even if the eGFR is normal [64]. In a cohort study conducted in Stockholm, Sweden, a fourfold increase in UACR was associated with a 3.08-times higher risk of ESRD, whereas a fourfold decrease in UACR was associated with a 0.34-times lower risk of ESRD, compared with stable UACR; these associations remained consistent regardless of T2DM, hypertension, or change in eGFR [65]. Other studies have suggested that, in addition to change in eGFR, change in albuminuria is another significant predictor of the future risk of CKD in patients with T2DM [66,67,68,69].

The panel recognized that albuminuria is prognostic of development of proliferative retinopathy. An 8-year prospective cohort study of a Chinese population with T2DM showed that elevated UACRs at baseline were associated with a significantly higher risk of developing proliferative retinopathy (31–300 mg/g: HR, 3.202; >300 mg/g: HR, 6.652), and that, after adjustment for baseline values, higher mean UACRs during the follow-up were still associated with a significantly increased risk of new-onset proliferative retinopathy (31–300 mg/g: HR, 2.344; >300 mg/g: HR, 4.193) [70].

Statement 12: ARBs are the favored regimens for the treatment of albuminuria in patients with T2DM and hypertension. 

The panel recommended ARBs for the treatment of albuminuria in patients with T2DM and hypertension, based on their efficacy and tolerability. As mentioned in previous statements, ARBs have demonstrated robust CV and renal benefits. They are effective in long-term BP lowering, with a 21% reduction in the risk of stroke compared with other antihypertensives [48,49]. Real-world patients on ARB-based therapy had a significantly higher BP attainment rate and fewer CV events than those on non-ARB-based therapy [50]. The efficacy of renal protection of ARBs was proven in meta-analyses [51,52], and they were shown to significantly reduce the risks of ESRD and doubling of the serum creatinine level by 23% and 40%, respectively, among T2DM patients with albuminuria [52]. A randomized trial of patients with T2DM showed that ARBs were associated with significantly reduced urinary excretion of angiotensinogen in participants overall (−5.4% vs. −1.7% at week 24), and lower albuminuria in participants with high-normal albuminuria (–4.8% vs. −4.3% at week 24), compared with treatment with a direct renin inhibitor [53]. In addition to efficacy, the tolerability of ARBs is promising, with a significantly lower risk of cough compared with ACEIs [16].

Part IV: Implications of albuminuria for CV/renal events and treatment choices

Statement 13: For the treatment of albuminuria in patients with hypertension and T2DM, it is preferred to use the ARBs that are supported by most available evidence in reducing the risk of doubling of serum creatinine and renal impairment.

International evidence showed that ARBs and ACEIs have similar efficacy in treating albuminuria in patients with T2DM and diabetic nephropathy [71,72]. However, as mentioned in a previous statement, the panel preferred ARBs due to a more favorable tolerability profile.

Landmark studies on renal outcomes of ARBs were reviewed (Table 2) [73,74,75,76,77,78]. Notably, only irbesartan [74,75] and losartan [76] were shown to significantly reduce the risk of progression of nephropathy in hypertensive patients with T2DM. In the IRMA-2 study of hypertensive patients with T2DM and microalbuminuria, irbesartan was associated with a significantly reduced risk of progression to diabetic nephropathy compared with placebo (300 mg: HR, 0.30; *p* < 0.001; 150 mg: HR, 0.61; *p* = 0.08) [74]. The IDNT study showed that, among hypertensive patients with T2DM and nephropathy, irbesartan at 300 mg significantly reduced the risk of doubling of serum creatinine by 37% and 33% compared with amlodipine 10 mg and placebo, respectively [75]. A similar study, RENAAL, showed that losartan significantly reduced the incidence of doubling of serum creatinine by 25% and incidence of ESRD by 28% compared with placebo [76]. Other ARBs may improve proteinuria, but with no renoprotective endpoints demonstrated. Considering the available evidence, most of the panelists prescribe irbesartan or losartan for the treatment of albuminuria in patients with hypertension and T2DM. 

Statement 14: Although the treatment of albuminuria in normotensive patients with T2DM remains uncertain, ARBs with proven renoprotective effects may be prescribed.

The panel recognized that whether and how to treat albuminuria in normotensive people with T2DM remain uncertain. A small prospective follow-up study of normotensive participants with T2DM and microalbuminuria in the RENAAL trial showed that those with rapid progression of albuminuria were at highest risk of reaching the endpoint of a composite of death, CV disease, cerebrovascular events, and peripheral artery disease, whereas those with a ≥30% reduction in albuminuria were at lowest risk, suggesting that a reduction in albuminuria may provide cardioprotection for people with T2DM and microalbuminuria, independent of BP [79]. A post hoc analysis of RENAAL demonstrated that, in patients with hypertension and diabetic nephropathy who reached the SBP target, a reduction in albuminuria remained associated with a lower risk of ESRD, exhibiting a BP-independent renoprotective effect [80]. Drawing from the preliminary evidence, the panel suggested that an ARB (irbesartan or losartan) may be prescribed for the management of albuminuria in normotensive people with T2DM, to reduce the risk of CV and renal events, along with monitoring for hypotension. To reduce the risk of hypotension, the ARB could be initiated at a low dose and taken at bedtime, with up-titration to the maximum tolerated dose, considering that the efficacy of a low-dose ARB in treating albuminuria may be suboptimal [74]. A sodium–glucose cotransporter-2 inhibitor or a mineralocorticoid receptor antagonist can be added if the UACR remains >30 mg/g after treatment with a full-dose ARB [81]. 

Part V: Roles and tools of screening for microalbuminuria

Statement 15: Regular screening for microalbuminuria in people with T2DM or hypertension is recommended for the monitoring of disease progression, assessment of treatment responses, and guidance in treatment decision-making.

Microalbuminuria or proteinuria is an independent risk factor for the decline in renal function, as well as increased CV morbidity and mortality [82,83]. A prospective 8-year study of patients with T2DM conducted in the UK showed that those with abnormal albumin excretion had significantly higher risks of all-cause mortality (odds ratio [OR], 1.47; *p* = 0.02) and vascular death (OR, 1.70; *p* = 0.009) than did those with normal albumin excretion, with the differences observed at all levels of abnormal albumin excretion, from just outside the normal range (10.6–29.9 µg/min) to microalbuminuria (≥30 µg/min) [82]. An analysis of the IDNT study showed that the risk of kidney failure doubled for each doubling of baseline proteinuria level (HR, 2.04; *p* < 0.001) and that each halving of proteinuria with treatment reduced the risk of kidney failure by more than half (HR, 0.44; *p* < 0.001), confirming the prognostic value of baseline proteinuria and the importance of therapy for proteinuria reduction in people with T2DM [83].

However, microalbuminuria is often underdiagnosed and undertreated in people with T2DM or hypertension. A large meta-analysis that included people with diabetes or hypertension showed that the rate of testing for UACR was low (35.1% in diabetes and 4.1% in hypertension) and largely unrelated to the predicted risk of albuminuria (median prevalence, 32.1% in diabetes and 21.8% in hypertension) [84]. A study conducted in public primary care clinics in Hong Kong demonstrated that therapeutic inertia (defined as failure to initiate or intensify ACEI/ARB treatment to control proteinuria) was present in 40% of cases of proteinuria among patients with T2DM (94% with concomitant hypertension) [85]. The panel recognized the importance of early detection and treatment of albuminuria, and, to optimize patient care, recommended regular screening for the condition in people with T2DM or hypertension [86].

Statement 16: Considering the balance between convenience and accuracy, a spot urine test for the measurement of UACR is the favored screening tool for microalbuminuria; to confirm a diagnosis, two of three UACR tests performed within a 3- to 6-month period should be abnormal.

The panel discussed the advantages and limitations of common screening tools for albuminuria, which include a 24-h urine collection, a urine dipstick test, and a spot urine test. A 24-h urine collection is expected to provide a more accurate result by addressing within-a-day and day-to-day fluctuations of albumin levels; however, the practical difficulties and poor compliance, especially among older people, often contribute to inaccuracy [87,88,89]. A urine dipstick test is a convenient method to assess the development of albuminuria or microscopic hematuria at routine follow-up visits, but one with higher sensitivity to macroalbuminuria than to microalbuminuria [90]. 

The panel noted that a spot urine test is the standard tool with which to balance convenience and accuracy [13,91,92], and that UACR, instead of urine albumin concentration, should be measured, because it demonstrates a stronger association with future renal events in people with T2DM and nephropathy [93]. To make a diagnosis of albuminuria, a positive spot urine test should be confirmed with one or two repeated measurements within 3–6 months, with consideration given to the factors that may temporarily affect UACR, including infections, dehydration, certain medications (e.g., COX-2 inhibitors and non-steroidal anti-inflammatory drugs), and vigorous exercise [64,81,86]. To reduce day-to-day variations in UACR results, spot urine samples should be collected at the same time in the morning for every test.

Statement 17: In patients with T2DM or hypertension, UACR and eGFR should be assessed at least annually to monitor disease progression and treatment response.

The panel pointed out that elevated UACR and low eGFR are both associated with an increased risk of CKD progression and are synergistic [64,81]. Both parameters should be monitored annually in stable patients and more frequently in high-risk patients, based on medical history, underlying cause of CKD, and previous data on UACR and eGFR [64,81].

## 5. Limitations

Several limitations of these consensus statements should be noted. Although the panelists mainly focused on the CV and renal health aspects of patients with hypertension and T2DM, both conditions can affect a variety of organs affected by different complications, such as non-alcoholic fatty liver disease [94,95,96], for which the screening strategy and treatment approach are not covered in this manuscript. These should be addressed by specialists in gastroenterology and hepatology. Additionally, it should be noted that a wide range of relatively new agents, such as sodium–glucose cotransporter-2 inhibitors, glucagon-like peptide-1 agonists, angiotensin receptor–neprilysin inhibitors, and aldosterone receptor antagonists, offer well-established CV and renal benefits in patients with T2DM or hypertension. Discussion of these agents was not included in this manuscript because their primary indications (i.e., hyperglycemia, heart failure, or CKD) are not hypertension. The panelists targeted the management of hypertensive patients with T2DM and thus mainly discussed the effects of commonly-prescribed antihypertensive drugs. Indeed, the effects of the above agents have been addressed by other experts from Hong Kong and mainland China [97,98,99].

## 6. Conclusions

Considering the real-life manifestation and the prognostic value, home BP (measured by a validated monitor for a sustained period) is as important as office BP in treatment decision-making for the management of hypertension. In general, antihypertensive drug treatment, preferably an ARB with proven cardioprotective and renoprotective effects, should be initiated for people with T2DM and hypertension, with the aims of achieving office BP < 130/80 mmHg (home BP < 125/75 mmHg) and controlling albuminuria. Albuminuria deserves more attention among physicians and patients. It is an independent risk factor for CV events, kidney failure, and all-cause mortality, as well as an important therapeutic goal for people with T2DM or hypertension. Albuminuria should routinely be measured in terms of UACR using spot urine testing in people with T2DM or hypertension.

## Figures and Tables

**Table 1:** Consensus statements.

Discussion Area	No.	Consensus Statement
Part I: Blood pressure (BP) targets based on cardiovascular (CV)/renal benefits	1	Home BP, in addition to office BP, should be considered for guiding treatment; home BP is generally considered 5 mmHg lower than office BP.
	2	In patients with type 2 diabetes mellitus (T2DM), hypertension is diagnosed when office BP is ≥140/90 mmHg and home BP is ≥135/85.
	3	In patients with T2DM and hypertension on antihypertensive drug treatment, the targets should be <130/80 mmHg for office BP and <125/75 mmHg for home BP.
	4	In patients with myocardial infarction (MI), other acute coronary syndromes, or stroke, the targets should be <130/80 mmHg for office BP and <125/75 mmHg for home BP.
	5	In patients with heart failure or chronic kidney disease (CKD), the targets should be <130/80 mmHg for office BP and <125/75 mmHg for home BP.
	6	In patients aged ≥ 80 years, the targets may be <140/90 mmHg for office BP and <135/85 for home BP.
Part II: Management of isolated systolic (ISH)/diastolic (IDH) hypertension	7	Both ISH (defined as systolic BP ≥ 140 mmHg and diastolic BP < 90 mmHg) and IDH (defined as systolic BP < 140 mmHg and diastolic BP ≥ 90 mmHg) should be treated to reduce the risks of MI, stroke, heart failure, and CV mortality.
Part III: Roles of angiotensin II receptor blockers (ARBs)	8	In patients with T2DM and hypertension, ARBs are the preferred antihypertensive drug regimens, considering their proven efficacy in reducing BP, as well as urine protein levels, the risk of end-stage renal disease (ESRD), and progression of nephropathy.
	9	For the treatment of hypertension, ARBs are preferred over angiotensin-converting enzyme inhibitors (ACEIs), considering the frequent side effect of dry cough associated with ACEIs, and the lack of evident differences in the BP lowering efficacy between ARBs and ACEIs.
	10	Combination therapy with an ARB and a calcium channel blocker can be considered for patients with inadequate BP control.
Part IV: Implications of albuminuria for CV/renal events and treatment choices	11	Regardless of the presence or absence of T2DM or hypertension, albuminuria should be treated as early as possible, considering the significant and continuous association between the degree of albuminuria and the subsequent risks of major CV events, ESRD, and proliferative retinopathy; these risks are further elevated in hypertensive patients with T2DM and albuminuria.
	12	ARBs are the favored regimens for the treatment of albuminuria in patients with T2DM and hypertension.
	13	For the treatment of albuminuria in patients with hypertension and T2DM, it is preferred to use the ARBs that are supported by most available evidence in reducing the risk of doubling of serum creatinine and renal impairment.
	14	Although the treatment of albuminuria in normotensive patients with T2DM remains uncertain, ARBs with proven renoprotective effects may be prescribed.
Part V: Roles and tools of screening for microalbuminuria	15	Regular screening for microalbuminuria in people with T2DM or hypertension is recommended for the monitoring of disease progression, assessment of treatment responses, and guidance in treatment decision-making.
	16	Considering the balance between convenience and accuracy, a spot urine test for the measurement of urine albumin-to-creatinine ratio (UACR) is the favored screening tool for microalbuminuria; to confirm a diagnosis, two of three UACR tests performed within a 3- to 6-month period should be abnormal.
	17	In patients with T2DM or hypertension, UACR and estimated glomerular filtration rate should be assessed at least annually to monitor disease progression and treatment response.

**Table 2:** Landmark studies on renal outcomes of angiotensin II receptor blockers (ARBs).

Study	Participant	ARB (n)	Comparator (*n*)	Primary Outcomes	Main Results
ONTARGET [73]	Patients aged ≥55 with established atherosclerotic vascular disease or with diabetes with end-organ damage	Telmisartan 80 mg/day (8541)	Telmisartan/Ramipril combination 80/10 mg/day (8502) Ramipril 10 mg/day (8576)	Composite of dialysis, doubling of serum creatinine, and death	Composite primary renal outcome was similar with telmisartan (HR, 1.00; 95% CI, 0.92–1.09), but increased with combination therapy (HR, 1.09; 95% CI, 1.01–1.18; *p* = 0.037)
IRMA-2 [74]	Hypertensive patients with T2DM and microalbuminuria	Irbesartan 150 mg/day (195)/Irbesartan 300 mg/day (194)	Placebo (201)	Progression to diabetic nephropathy based on increases in proteinuria	Reduction of progression to diabetic nephropathy (irbesartan 300 mg HR, 0.30; *p* < 0.001; irbesartan 150 mg HR, 0.61; *p =* 0.08)
IDNT [75]	Hypertensive patients with T2DM and nephropathy	Irbesartan 300 mg/day (579)	Amlodipine 10 mg/day (567) Placebo (569)	Doubling of serum creatinine, development of ESRD, or death from any cause	Irbesartan reduced the incidence of doubling of serum creatinine vs. amlodipine (37% RR; *p* < 0.001) and placebo (33% RR; *p =* 0.003)
RENAAL [76]	Hypertensive patients with T2DM and nephropathy	Losartan 100 mg/day (751)	Placebo (762)	Doubling of baseline serum creatinine, development of ESRD, or death from any cause	Losartan reduced the incidence of doubling of serum creatinine (25% RR; *p =* 0.006) and incidence of ESRD (28% RR; *p =* 0.002) vs. placebo
MARVAL [77]	Patients with T2DM and microalbuminuria, with or without hypertension	Valsartan 80 mg	Amlodipine 5 mg	% Change in UAER from baseline to 24 weeks	Valsartan lowered UAER more effectively than amlodipine

CI, confidence interval; ESRD, end-stage renal disease; HR, hazard ratio; T2DM, type 2 diabetes mellitus; UAER, urine albumin excretion; RR, risk reduction.

## Data Availability

The authors confirm that the data supporting the findings of this study are available within the article.
